# A heuristic approach and heretic view on the technical issues and pitfalls in the management of penetrating abdominal injuries

**DOI:** 10.1186/1757-7241-18-40

**Published:** 2010-07-14

**Authors:** Tugba H Yilmaz, Brown C Ndofor, Martin D Smith, Elias Degiannis

**Affiliations:** 1 Department of Surgery, Baskent University, Izmir, Turkey; 2 Department of Surgery, Chris Hani Baragwanath Hospital, University of the Witwatersrand, Johannesburg, South Africa

## Abstract

There is a general decline in penetrating abdominal trauma throughout the western world. As a result of that, there is a significant loss of expertise in dealing with this type of injury particularly when the patient presents to theatre with physiological instability. A significant percentage of these patients will not be operated by a trauma surgeon but, by the "occasional trauma surgeon", who is usually trained as a general surgeon. Most general surgeons have a general knowledge of operating penetrating trauma, knowledge originating from their training years and possibly enhanced by reading operative surgery textbooks. Unfortunately, the details included in most of these books are not extensive enough to provide them with enough armamentaria to tackle the difficult case. In this scenario, their operative dexterity and knowledge cannot be compared to that of their trauma surgeon colleagues, something that is taken for granted in the trauma textbooks. Techniques that are considered basic and easy by the trauma surgeons can be unfamiliar and difficult to general surgeons.

Knowing the danger points and pitfalls that will be encountered in penetrating trauma to the abdomen, will help the occasional trauma surgeons to avoid intraoperative errors and improve patient care. This manuscript provides a heuristic approach from surgeons working in a high volume penetrating trauma centers in South African. Some of the statements could be considered heretic by the "accepted" trauma literature. We believe that this heuristic ("rule of thumb" approach, that originating from "try and error" experience) can help surgical trainees or less experienced in penetrating trauma surgeons to improve their surgical decision making and technique, resulting in better patient outcome.

## The Liver

On opening the abdominal cavity and encountering torrential hemorrhage that cannot be easily controlled by direct clamping or pressure, (e.g. extensive liver injuries, injuries to the aorta and its branches, injuries to the inferior vena cava etc) it is advised in the trauma literature to clamp the aorta below the diaphragm as it enters the abdominal cavity between the two cruras. This involves division of the lesser omentum, followed by traction of the lesser curvature of the stomach to the left. The abdominal esophagus is then dissected (sharp dissection of the peritoneum over the anterior aspect of the esophagus, followed by the creation of a groove between the sides of the esophagus and the two cruras with the use of pledgets). The esophagus is then encircled with the index finger and pushed towards the left. This brings into direct vision the anterior aspect of the proximal abdominal aorta which is then clamped with a vascular clamp. The above description is by itself tiring. Imaging doing the above dissection on a patient who does not have a recordable blood pressure and is exsanguinating in front of your eyes! To make things even worse, we all are well aware of how difficult and tricky it is to effectively clamp the aorta as it passes through the two cruras. The fact that posterior to the aorta are the bodies of the lower thoracic vertebrae, makes the clamp prone to slipping off, resulting only to partial occlusion of the lumen. To avoid this (although not always successful) it requires an assistant to control/hold the vascular clamp at all times. There are special T-shaped vascular clamps designed for occlusion of the aorta that work not only by occlusion of the aortic lumen but also by compressing the not occluded part, with the T part of the clamp pressed against the vertebral column. Unfortunately these clamps are usually not present in the standard laparotomy and vascular sets. Insertion of a vessel loop around the aorta at this area with the help of a right angled Lahey, although not easy, can save the day as it can give excellent proximal control. A cheap and effective alternative to the standard clamps has just been reported in the literature as used in South African trauma centers [1]. This "clamp" is a wooden spoon with convex arches cut from its base. It is used for occluding the aorta (or the IVC) by compressing these structures against the vertebrae, giving vascular control while leaving good surgical access. It is worth trying it. In our opinion, (as also expressed in the past by other trauma surgeons, but not favored in the recent trauma literature), patients with penetrating trauma to the abdomen presenting in theatre with a very low systolic blood pressure and massively distended abdomen, should initially be dealt with by a left anterior lateral thoracotomy and clamping of the aorta above the diaphragm. The application of a "prelaparotomy thoracotomy" avoids an otherwise difficult clamping of the aorta, contributes to the safeguard of the blood supply of the vital organs and results in partial control of the intra abdominal hemorrhage, maintaining an acceptable blood pressure and facilitating effective intraabdominal dissection for direct bleeding control [2-4]. There is no doubt that it adds to the patients postoperative morbidity but sometimes it is the only way in successfully getting the patient out of theatre alive.

Managing extensive liver injuries requires a well planned operative approach. The surgeon must be familiar with the various alternative approaches recommended in the literature in tackling a scenario fraught with hazards. As evidenced by the various methods, there is no "silver bullet" in dealing with major liver injury. At times, irrespective of all your efforts, you will find yourself desperate in theatre with your patient dying from uncontrolled hemorrhage. On opening the abdominal cavity and encountering a torrentially bleeding liver injury, it is paramount to try and control the source of bleeding using local pressure; this gives the anesthetist the opportunity to improve the patient's physiological parameters. A Cellsaver should always be available. Proceed with the Pringle's maneuver. This is achieved by occluding the extrahepatic portal triad, encircling it initially with a finger through the lesser omentum, and occluding it by the smallest Satinski clamp available, applied from left to right so that it stays as far away as possible from your operative field, if you decide to operate from the patient's right side. If access to the injury requires mobilization of the entire liver, you should move to the patient's left. From this position, it is easier to mobilize and lift the right lobe, almost to the level of the abdominal incision. After dividing the falciform ligament with diathermy, divide the right coronary ligament (if there is need) with scissors from its attachment to the diaphragm, by applying downwards traction to the right lobe. Be aware of the right hepatic vein that can be damaged, just as it enters the IVC, during the mobilization of the most medial aspect of the right triangular ligament. Insert your open left hand behind the right lobe and lift the liver towards the abdominal incision, as it rest on your hand and distal forearm. When this maneuver is accomplished (you hear a sucking noise as air is sucked behind the liver), insert several folded abdominal swabs, deep to the dorsal aspect of your distal forearm and hand, into the liver bed. Then remove your hand, leaving the liver to rest on the swabs, elevated anteriorly into the abdominal incision. Although uncommon, in the presence of an enlarged heavy liver, this maneuver may cause excessive pressure on the retrohepatic IVC further aggravating the haemodynamic instability. Take care not to in avertedly divide/tear the short anterior branches of the IVC as they enter the liver parenchymal. Unless they are bleeding, avoid touching them! It is wise to mention at this point that a trauma surgeon or a general surgeon, who deals with an extensive liver injury, does not have the expertise of a liver or a liver transplant surgeon. The aim of the whole exercise is primarily to control the bleeding. Liver resections or fancy maneuvers are not in the scope of practice of trauma surgery. We are so convinced of this issue that in discussing the management of liver injury with our junior doctors, we exaggerate by stating, that "the trauma surgeon does not need to know the segmental liver anatomy"!

In the situation when the hemorrhage is coming from the bullet tract, plug the tract. Many methods of plugging have been described in the literature, proof that none of them works in all cases. We have tried them all, with variable outcomes. Over the last few years, we have come to favor packing of the tract with Alginate (kaltostat). Kaltostat is hard, reasonably pliable and does not soften or dissolve in the presence of blood. It can indefinitely be left in situ, especially in cases where the tract is long and attempts to remove the kaltostat could lead to difficulty in controlling recurrent hemorrhage. After you plug the tract, if there is still some bleeding, pack the liver with abdominal swabs making sure that these are covered with opsite to prevent tearing the liver capsule on removal of the packs. The use of saline inflated "condoms" or Sengstaken-Blakemore tubes in control of bleeding from tracts, although impressive as an idea, are not easy to apply in the emergency situation. In the event of further persistent of bleeding from a tract, attempt intrahepatic haemostasis by performing a tractotomy. Divide the Glissonian capsule with diathermy and proceed along the tract using finger fracturing of the hepatic parenchymal. Keep in mind that tractotomy is feasible and safe only if the tract is superficial. Going through a significant volume of the parenchymal to access a bleeding point, will result in additional bleeding from the cut liver surface. Doing this in a patient who is already exsanguinating is absolute madness! [5]. Unfortunately our experience with the application of glues in a bleeding tract has never resulted in the control of haemorrhage.

The use of mattress sutures is useful in controlling bleeding from exposed liver parenchymal, particularly in areas that are difficult to access. The small areas of liver necrosis produced by the applications of haemostatic liver sutures rarely cause any problems. You can also use haemostatic glues in deep cavities by themselves or as an additive to other methods of haemorrhage control [6-8].

There are situations (luckily rare) in which you will have to consider selective hepatic artery ligation. What is not stated in the trauma literature is the difficult and time consuming procedure of dissecting and selectively ligating the right or the left hepatic artery, particularly in the presence of massive bleeding from the liver in an already physiologically unstable patient. This is a formidable task even in the non-trauma scenario. If you manage to dissect these arteries easily and fast, all well! But in desperate situations where dissection is not possible and the patient is dying, take a strong stitch on a large non cutting needle. Insert the stitch blindly at the hilum (where you expect the beginning of the intrahepatic course of the right or the left hepatic ducts to be), taking a big bite, aiming to encircle the bile duct and with it the artery, as the stitch on being tied cuts through the surrounding liver parenchymal. We believe that this is the only way of succeeding, quick and efficient ligation of any one of the two main branches of the common hepatic artery and offering to our patient a chance of survival under extremely adverse circumstances. Certainly, there is a high chance of the corresponding hepatic lobe becoming necrotic, but at least your patient is still alive! In the next few days the hepatobiliary surgeon can get involved if liver resection becomes necessary.

If you manage to control the bleeding after applying the various maneuvers, remove the Satinski clamp. If there is no further bleeding, transfer the patient to a high dependency unit. However, if on removal of the clamp bleeding resumes, reapply the Satinski and consider the presence of an anomalous arterial blood supply or injury of the hepatic venous or retrohepatic vena cava. Incise the lesser omentum, in search of an aberrant left hepatic artery arising from the left gastric artery. Ligate it if the arterial bleeding corresponds to its feeding area.

It is important to establish from the beginning of the operation if the bleeding is due to a retrohepatic inferior vena cava injury. This injury has a very high mortality rate and it has been shown that even in the best of hands, any attempt of repair can lead to dismal outcomes. Instead of attacking the vein head on, pack the area, and in most instances it will result in control of the bleeding especially if the injury is not extensive. It is important to identify this injury before dividing the hepatic ligaments. If you have already divided these ligaments, it will be very difficult to control the bleeding by pressure because the liver is not supported by its ligaments and floats inside the abdominal cavity making packing inefficient. Retrohepatic IVC or hepatic vein injuries that are not controlled with packing, have an extremely high mortality rate. The suggested atriocaval shunt that isolates the injury while it maintains the blood flow in the IVC by bypassing the injury site, is challenging to perform: just consider a tense inexperienced surgeon inserting a purse string in a flimsy atrial wall around a tube that has been inserted to bypass the IVC injury! We do not anymore practice retrohepatic IVC shunts as we have never had any survival. Instead of shunting, we have practicing total liver isolation with few survivals [9]. We feel that although theoretically, an IVC shunt is much more technically difficult than liver isolation and with no better results in our hands or as well as in the international literature.

## The Duodenum

Mobilization of the whole duodenum is mandatory for the identification of duodenal injuries. To perform the Kocher maneuver, it is easier to stand on the patient's left side. After you mobilize the hepatic flexure and the proximal third of the transverse colon, with your scissors, you divide (while applying traction on the second part of the duodenum) the peritoneum, laterally to the second part of the duodenum, together with the underlying lateral duodenal ligament. Most people tend to forget the presence of this ligament which attaches the second part of the duodenum to Gerota's fascia, and often try to mobilize the duodenum by rotating it medially while the ligament is still intact. This can result into tearing of its wall. After dividing these two structures at the same line with your scissors, insert your index finger under them and continue this line of sharp dissection proximally to the foramen of Winslow and distally to the superior mesenteric vein as it crosses the third part of the duodenum. At the end of this maneuver, you should be able to palpate the aorta posterior to the pancreas and fully visualize the anterior and posterior aspects of the second and third part of the duodenum, as well as the head and uncinate process of the pancreas.

To expose the posterior aspect of the first part of the duodenum and the medial aspect of the second part, enter the lesser sac by dividing the gastrocolic ligament. To access the third and forth parts of the duodenum, mobilize the right colon (including the hepatic flexure) from right to left and elevate the right colon and small intestine. Then mobilize the small bowel by sharply incising the retroperitoneal attachments from the lower right quadrant to the ligament of Treitz. Remember to replace the small bowel in the abdominal cavity with great care at the conclusion of the operation to avoid rotation volvulus.

In the majority of cases, primary repair of the duodenum is indicated, particularly when the time interval between the injury and the operation is short. Do not forget to insert a drain in the vicinity of the repair. In a minority of cases, if you feel that there is an increased likelihood of suture line dehiscence, fashion a pyloric exclusion as an adjunct to the primary repair [10]. We do not advocate the triple ostomy (gastrostomy to decompress the stomach, retrograde jejunostomy to decompress the duodenum and antrograde jejunostomy to feed the patient) as we try to avoid the creation of too many anastomosis. The inefficiency of the retrograde jejunostomy in decompressing the duodenum, and the scenario of feeding tubes falling out, are well known and documented [11]. Consider fashioning the feeding jejunostomy at the initial laparotomy in patients with duodenal injury and extensive abdominal trauma (abdomen trauma index greater than 25). Never do a truncal vagotomy, and we agree with reports that stomal ulceration is not an issue (but acknowledge the limited follow up in penetrating trauma patients worldwide). It would have been interesting to understand what happens to the gastrojejunostomy after the reversal of the pyloric exclusion. As a mucosa to mucosa anastomosis this should never close but on the other hand it seems as if it stops functioning. The suture used for the pyloric closure makes no difference as all pyloric exclusions will open within a few weeks [12].

## The Pancreas

Concerning penetrating injury of the pancreas; if a parenchymal injury is noted, it is important to determine the integrity of the main pancreatic duct keeping in mind the intra operative criteria introduced by Heitsch et al which includes: direct visualization of ductal violation, complete transection of the pancreatic parenchymal, laceration of more than half the diameter of the pancreas, central perforations and severe maceration [13]. Proceed to full mobilization of the injured area. Observe the distal pancreas by partially opening the greater omentum and if there is anything suggesting injury, open the whole lesser sac by detaching the greater omentum from the transverse colon along the bloodless line, to expose the full length of the lesser sac. If you suspect that the injury may involve also or only the posterior aspect of the body and tail of the pancreas, incise the avascular peritoneal attachment of the transverse mesocolon to the pancreas and expose its inferior border for proper visualization of any injury suspected at the posterior aspect of the body and tail of the pancreas. Subsequently, lift the pancreas upwards by blunt dissection with your fingers in the retropancreatic space, this allows you access to the upper border of the pancreas (you should also incise the peritoneum superficial to your fingers along the upper border of the pancreas).

The splenic vein is closely adherent to the posterior aspect of the pancreas and your dissection should proceed posterior to the splenic vein. Perform a cephalad rotation of the pancreas that will allow you inspection of the posterior surface and bimanual palpation. This maneuver can be performed safely as long as the initial sharp dissection is properly completed. A few retropancreatic vessels may bleed, but this can easily be controlled by local pressure. If after mobilization of the pancreas, you feel that distal pancreatectomy is necessary, you should ligate the splenic artery and vein 1--2 cm proximal to the injury site. Continue the mobilization of the pancreas for 1--2 cm to the right of the site of the proposed resection. Then apply a soft-bowel clamp and divide the parenchymal with sharp dissection or electrocautery. Gradually release the soft-bowel clamp so that you can identify the two pancreaticoduodenal arteries, and then overrun them with a vascular stitch. Our experience is that in many cases it is also possible to identify the minute pancreatic duct, in which case it is advisable to occlude it with a vascular stitch. Close the pancreatic stump by performing overlapping interrupted mattress stitches using polypropylene or silk. This technique of mattress sutures can by itself achieve parenchymal closure as well as adequate homeostasis and occlusion of the pancreatic duct, although we prefer to occlude the arteries and the duct separately if possible, and then continue with the mattress sutures [14]. Resection of the body of the pancreas can also be achieved with a linear stapler. Initially we used both hand-sewing and stapling techniques, without significant difference in outcome for approximately 70 patients who underwent distal pancreatectomy for gunshot injury to the distal pancreas [15]. However, over the last 10 years we have observed that the stapling technique has been unsatisfactory as in a significant percentage of cases, the stapled line required reinforcement with sutures. We found the GIA staplers absolute (completely crushing the soft and thin pancreatic tissue and a lot of times failing to hold the tissue or occlude the pancreatic duct with a high incidence of pancreatic fistulas. In our hands TA staples have better results as the pressure exerted on the pancreatic tissue is not standard, as with GIA, and can to a certain extend be controlled by the surgeon. In our institution, the best result in achieving control of the pancreatic stump is when we do not use staplers. This observation (which, to our knowledge, has not been reported by other authors) has prompted us to use only the hand-sewing technique. A structured review of our results, as well as further reports of extensive studies, are required to justify our caution in avoiding stapling in cases where the hand-sewing technique is not contraindicated. We treat the majority of penetrating injury to the head of pancreas with drainage and only rarely elect to do pancreaticoduodenectomy, except when it is unavoidable and in fact this is usually when most of the dissection has been done by the mechanism of injury. Remember that, though few in gross numbers, more patients' lives are eventually saved by drainage, total parenteral nutrition and meticulous overall care, than by a desperate pancreaticoduodenectomy in a marginal patient [16]. Perform pancreaticoduodenectomy only as two-stage procedure. After the initial damage control operation and achievement of homeostasis, you should staple off the stomach, jejunum and control the pancreatic stump. Ligate or drain the common bile duct. Complete the anastomosis at reoperation within the next 48 hours, when the patient is stable [17]. There are two main differences between performing a pancreaticoduodenectomy in the clinical setting of trauma and that of cancer. First, in trauma surgery it is not necessary to remove the uncinate process. This simplifies the procedure, as you can operate away from the superior mesenteric vein. Second, the gall bladder is not removed in a trauma case as it can be used for biliary-enteric reconstruction in the presence of a small diameter common bile duct. There is controversy regarding the management of the pancreatic stump after pancreaticoduodenectomy. A soft, normal pancreas with a normal main duct is found in the great majority of trauma cases. This generates technical difficulties with ensuing complications. In an attempt to tackle this problem, the pancreatic stump has been managed in various ways, including ligation of the pancreatic duct and pancreaticoenteric or pancreaticogastric anastomosis. Although ligation of the pancreatic duct in the non-trauma situation has been associated with a significantly higher fistula rate when compared with anastomosis, the mortality rate is not significantly different. The experience in trauma is limited, and pancreatic duct ligation has been advocated as a technique available when faced with an unstable patient unable to tolerate further operations. The long-term risks of beta cell function insufficiency among young trauma patients are disputed. Pancreatico-gastric and pancreatico-enteric anastomosis have been reviewed, with the former advocated as an exceptionally safe procedure. On the other hand, its superior safety compared with other conventional techniques has yet to be proved, particularly with the declining trend in the incidence of pancreatic fistula and related mortality following pancreaticojejunostomy. Total pancreatectomy has been advocated to obviate the consequences of a leaking stump, but this can create an endocrine cripple with a brittle endocrine status. Any Roux-en-Y anastomosis to incorporate the injured area in the head of the pancreas at the time of injury is ill advised because of the high risk of anastomotic breakdown Injury to the neck, body or tail of the pancreas with major lacerations or transections and associated duct injury is best treated by distal pancreatectomy and splenectomy. It has been suggested that the resection margin should be anastomosed to a Roux en Y loop, to prevent the development of a pancreatic fistula. This procedure is time consuming and therefore inappropriate for patients with multiple injuries. Even if the patient is physiologically stable, an anastomosis between a normal soft pancreatic remnant and a Roux-en-Y loop of bowel is unsafe and is likely to leak [18]. Now, what about splenic preservation in distal pancreatectomy? This is something worth keeping in mind. This is technically not challenging, but quite tedious and the patient must be physiologically stable with no other time consuming injuries present, which is uncommon in penetrating pancreatic trauma.

## The Spleen

Significant injury to the spleen necessitates splenectomy except in special circumstances. The majority of patients with penetrating injury to the spleen are adults. The significance of splenectomy in this age group is controversial with respect to the incidence of Overwhelming Post Splenectomy Infection (OPSI) [19]. Consider splenic preservation in adults in malaria infested areas, as it has been suggested that its removal is associated with an increase in mortality from complications of malaria. Control the bleeding in minor injuries by application of pressure or haemostatic agents. In the trauma literature, it is widely stated that major injuries to the spleen can be repaired with the use of sutures, mesh pouches or by performing partial splenectomy. As practicing surgeons we find the above difficult to perform and misguiding to younger colleagues. Attempts to preserve the spleen in the presence of major splenic injury by suturing or amputating the injured part, is discouraged in our institution as it usually still requires a splenectomy after significant hemorrhage and time loss by the frustrated surgeon. If your patient is physiologically stable and you decide to attempt repair of the splenic injury, it is absolutely necessary to mobilize the spleen and elevate it to the level of the abdominal incision. So proceed with division of the corresponding ligaments and ligation of the short gastric arteries. Then apply a Satinsky clamp at the hilum.

Try to repair the amputated spleen after completely removing the amputated part, (if it is still attached to the spleen) control the hemorrhage from the splenic parenchymal by interrupted horizontal mattress sutures. Using a curved needle sometimes causes significant bleeding from the spleen through the circular movement of the needle and most of the times the needle is too short to enter and exit the opposite surfaces of the spleen. Instead of using an ordinary needle, use a gauge 22 lumbar puncture needle. Put the lumber puncture needle through a pledget and then advance the needle through and through from the anterior to the posterior surface of the spleen. Then, as the tip of the needle passes through the posterior surface of the spleen, thread another pledget over it. Insert a thread through the lumen of the lumbar puncture needle in the direction from its bevel to its tip end and remove the needle. Reinsert the needle about 1 cm laterally, again in an anterior to posterior direction, repeating the same procedure with the pledgets, this time inserting the thread that has previously been passed through the spleen, in the lumen of the needle in opposite direction-from the tip of the needle to the bevel. Remove the needle and tie the two ends of the thread and you have your first interrupted horizontal mattress stitch on pledgets. Continue the same process till the mattress suture involves the whole raw surface of the spleen. Haemostatic glues on the sutured raw surface can improve outcome of the above technique.

## The Inferior Vena Cava (IVC)

On encountering significant bleeding originating from infrahepatic (suprarenal and infrarenal) inferior vena cava (IVC), the easiest way of controlling the hemorrhage is by compressing the vein proximally and distally to the injury site, using swabs on a stick or the wooden spoon clamp as mentioned above. This way, you succeed in having a "dry" as possible operative field. This is what the books say. In our practice we compress the IVC proximally and distally by the short limb of a Langenberg retractor, and we have found it to be more efficient than using the swabs on the stick. Some colleagues suggest the use of vessel loops. Theoretically, if these are inserted carefully with a right angled Lahey, this should do the trick. We do not have any experience on that, but it's worth keeping in mind and, why not, try it! Now grasp the two sides of the defect with Babcock clamps, lift them and occlude the IVC defect by applying a Satinski clamp along the IVC, underneath the Babcocks. Keep in mind that although this technique is the best available and sounds elegant and efficient, it is challenging, as the initial occlusion of the lumen by pressure with the swabs on a stick, never completely controls the bleeding. The use of a vascular sucker and coordinated action by members of the surgical team is of paramount importance. You must be in control of the situation, and make sure that you discourage any unnecessary movement by the assistants until the Satinski is applied, completely controlling the bleeding and bringing relief to the whole team. Remember that the application of the Satinski, by itself, is not without risks. Due to the anatomical depth of the injury, it is desirable to keep your assistant's hands outside the operating field as much as possible; therefore a large Satinski is usually applied on the IVC. By so doing, the assistant holds the handle of the clamp with his hand outside the operating field. Two things can then go wrong; firstly, ripping further the already injured IVC wall. This is "successfully" achieved by the assistant applying upwards traction on the large clamp in an attempt to help the surgeon while he is repairing the defect. Secondly, by the assistant leaving the Satinski to float in the abdominal cavity, in attempting to use his hands and take the viscera away from the operative field, "facilitating" again the repair by the surgeon. The weight of the free-floating Satinski can by itself tear the IVC. In the lacerated IVC, the edges of the defect contract, due to the elastic fibers of the wall, making the identification and the exact grasping of the edges with the Babcocks difficult. Furthermore, the application of the Satinski includes a significant portion of the wall within the limbs of the clamp. Therefore at the end, there is not enough "cloth" left to repair the vein with a continuous stitch, above the limbs of the clamp. Because of that, it is not surprising that successful repair of the IVC results in significant stenosis of the vein. Do not worry about it, for little can be done. The natural history of this is that, in most cases the stenotic part thromboses and later recannalises within the next few months, during which time the body tackles the problem by collateral vessels. Taking into consideration the effort, the loss of time and the additional loss of blood during the repair of the vein, it is worth considering ligation of the IVC in the patient with significant IVC injury and physiological instability. It is widely thought and mentioned in the literature that although ligation of the infrarenal IVC is an acceptable method of bailing out the patient (and the surgeon), it is forbidden in the case of the suprarenal IVC, as it results in loss of both kidneys. This was applicable in the past, as supporting life in a patient with non functional kidneys was problematic. Nowadays, although it is still desirable to repair the suprarenal IVC, you should not hesitate to ligate it if this is necessary to save the patient's life. With the patient alive, there are ways and means for sustaining him for long periods and even consider him for kidney transplant at a later stage. Ligation of the suprarenal and infrarenal IVC is surprisingly well tolerated by the majority of patients. Most of them develop minimal edema of the lower limbs, responding to the application of graded compression stockings. Lack of significant symptoms and signs after three months, is the rule. In a few cases, we have observed the formation of a significant amount of ascitic fluid post IVC ligation, up to a volume of 5 liter/day that has been draining through the abdominal drain sites. Surprisingly, in all these cases, drainage ceased completely within a week from the day of operation. It is advised that in the case of damage of the posterior wall of the IVC, this should be repaired by rotating the vessel (which is very difficult, as you have to rotate the IVC against the lumbar veins with possible resultant further damage) or by incising the anterior wall of the IVC, repairing the posterior wall, and then closing the anterior defect. Although the later sounds "sound" in description and impressive in the sketches of operative books, it is again not without the risk of making things worse. A posterior wall defect sometimes can be controlled by application of pressure on the IVC (anterior to posterior pressure), this has been shown feasible in laboratory animals, but is this enough to practice in humans? Consider ligating the IVC proximally and distally to the defect, instead of embarking on challenging and most times unsuccessful repairs that can only lead to physiological deterioration of the patient. If bleeding from the lumbar veins still continues after ligation of the IVC, then open continuously the whole anterior wall of the vein that is included between the two ties and over sew the bleeding lumbar veins from inside the IVC [9]. Mentioning all the above ligation of the IVC, it does not mean that this is free of complications and therefore preferable to repair. Extensive proximal thrombosis can lead to death and resistant distal thrombosis can lead to debilitating post phlebitis syndrome.

## Epilogue: "There are many ways to skin a cat!"

The present manuscript on thoughts, technical issues and pitfalls in penetrating injury to the abdomen by no means covers the full extent of the subject. It also has a very personal character in managing certain abdominal penetrating injuries. There is no doubt that a lot of experienced trauma surgeons from around the world will have a different heuristic approach in encountered problems and in some cases more appropriate. Therefore we hope that the heuristic approach of this manuscript will hopefully be the catalyst for a stimulating discussion/debate and further manuscripts of this type in all kinds of trauma. This will help the less experienced of us in to improve our decision making and surgical techniques, resulting in better patient outcome.

## Competing interests

The authors declare that they have no competing interests.

## Authors' contributions

TH: Idea on comparing the practice of trauma at Chris Hani Baragwanath Hospital and international practice. Literature review. Contribution in writing manuscript.

BC: Contribution in writing manuscript.

MD: Contribution in writing manuscript

ED: Contribution in writing manuscript and overall supervision.

All authors have read and approved the final manuscript.
